# A Comprehensive Evaluation of Studies on the Adverse Effects of Medications in Australian Aged care Facilities: A Scoping Review

**DOI:** 10.3390/pharmacy8020056

**Published:** 2020-03-31

**Authors:** Haider Qasim, Maree Simpson, Yann Guisard, Barbora de Courten

**Affiliations:** 1 School of Biomedical Science, Charles Sturt University, Orange 2800, NSW, Australia; masimpson@csu.edu.au; 2 Faculty of Science, Charles Sturt University, Orange 2800, NSW, Australia; yguisard@csu.edu.au; 3 Faculty of Medicine, Monash University, Clayton 3168, VIC, Australia; barbora.decourten@monash.edu

**Keywords:** scoping review, ADR assessment, elderly, aged care facilities, medications monitoring, nursing home, drug review

## Abstract

**Aim:** this scoping review was designed to identify studies that assess adverse drug reactions (ADRs) for older people in Australian aged care facilities. This review critically evaluates each published study to identify the risk of, or actual, adverse drug events in older people. Inclusion criteria: this review considered any clinical studies that examined the adverse effects of medications in older people who were living in aged care facilities. This review considered qualitative studies, analytical studies, randomized controlled trials (RCTs), descriptive cross-sectional studies, and analytic observational studies that explored the use of medications and their adverse effects on older people in clinical settings (including aged care facilities). **Methods:** an initial search of the PubMed (United State National Library of Medicine), OvidSP, EBSCOHost, ScienceDirect, Wiley Online, SAGE, and SCOPUS databases, with full text was performed, followed by an analysis of the article’s title and abstract. Additionally, MeSH (Medical Subject Headings) was used to describe the article. The initial round of the database search was based on inclusion criteria from studies that assessed tools or protocols aiming to identify the adverse effects of medications on the elderly population suffering chronic conditions or multiple co-morbidities. Two reviewers screened the retrieved papers for inclusion. The data presented in this review are in tabular forms and accompanied by a narrative summary which aligns with the review’s objectives. **Results:** seven studies were identified, and the extracted data from these studies were grouped according their characteristics and the auditing results of each study. **Conclusion:** it would be beneficial to design a comprehensive or broadly adverse drug reaction assessment tool derived from Australian data that has been used on the elderly in an Australian healthcare setting.

## 1. Introduction

In 2050, there will be over one million Australians living in aged care facilities [1]. Approximately half of this population is predicted to have cognitive impairment, while the remainder are likely to suffer from one or more chronic diseases such as depression, diabetes, cardiovascular diseases, neurodegenerative diseases and rheumatological conditions [1]. The adverse effects of medications can complicate the management of multiple chronic diseases, which often makes it challenging to follow clinical guidelines [2]. Numerous tools and protocols are available to assess the side effects of medication in aged care facilities. However, they are often specific to certain medical conditions and do not provide a comprehensive assessment of the medication’s side effects [3]. Adverse drug reactions (ADRs), defined as unwanted harmful reactions, result from an intervention related to the use of one or more medicinal products [3] ADRs usually require prevention, specific treatment, the alteration of a dosage regimen, or drug discontinuation [4]. One of the major causes of ADRs arises from inappropriate prescribing cascades, whereby a new medication is given to manage the adverse effect of that inappropriate drug, thus exposing the patient to continued risks of ADRs from culprit drugs and the newly prescribed drug [5]. In some cases, the adverse drug reaction (ADR) symptoms may be incorrectly interpreted as a primary diagnosis rather than as side effects of the medications [6]. This added complication in distinguishing between drug-induced symptoms and definitive medical conditions may result in additional medications being prescribed [6]. 

A scoping review was selected over a systematic review because the concepts of a scoping review are ideal to determine the depth and breadth of a body of literature on a given topic, such as the adverse effect of drugs in older people; it also gives a clear indication of the volume of the literature and studies that are available on this topic and provides more detail to the focus. A scoping review is a useful approach to examine each piece of evidence in detail and concerns more specific questions and gives more illustration about the inclusion and exclusion criteria. A scoping review is applicable in our topic to identify: (1) the types of available evidence about the effects of medications on older people; (2) to clarify key concepts and definitions in the published papers; (3) to examine how the research or study was done or conducted on our topic; (4) to identify the characteristics of each included study; (5) and to identify and analyse the gap needing to be covered in clinical practice. 

This scoping review searched the current academic literature for ADR assessment in Australian aged care facilities to identify studies that were summarized, and a critical evaluation was undertaken for each study. We conducted our scoping review between 2017 and 2020, and papers published up to February 2020 were included, with MeSH terms updated to reflect narrower subheadings that were added since March 2017. Seven databases where searched: PubMed, OvidSP, EBSCOHost, MEDLINE, ScienceDirect, Wiley Online, SAGE, and SCOPUS. The inclusion criteria were established and were informed by the PICO model: No restrictions were set concerning the elderly in Australian health care (P), interventions/tools for monitoring (I), assessment of the adverse effects of medication tools (C) and medication management in nursing homes or hospitals (O) which were used to frame the data extraction. In addition to PICO, the following study selection criteria were formulated: randomized controlled trials (RCTs) and only full-length articles were considered for inclusion in this review. Two reviewers (HS and YG) independently selected titles and abstracts and the corresponding full text articles were included in this scoping review. Any discrepancies in judgment (whether the article was included or excluded) were discussed in order to reach a consensus (MS and/or BD) about final inclusion. 

**Aims and review’s questions**

The aim of this scoping review was to establish which tools or protocols are being used in Australia to determine the adverse effects of medications in older people living in Australian aged care facilities. More specifically, the review questions were:

1. What are the types of adverse effects identification tools currently used in Australian health care settings (aged care facilities and hospitals)?
2. What evaluation outcome measures have been reported for the tools in primary care settings?
3. Does the tool or protocol minimise the adverse effects of medications without compromising the benefits of medications?
4. Do the tools improve patients’ clinical outcomes by identifying inappropriate medication prescribed or medication errors?
5. Do the tools or protocols support multi-disciplinary interventions through optimising day-to-day patient care?

**Inclusion criteria**

The selected studies were based on the following:

- The study was intended for patients aged 65 years or older;
- The study included older patients who experienced adverse effects of medications;
- The study included older patients suffering from the adverse effects of polypharmacy and living in aged care facilities or admitted in hospitals;
- The study investigated tools that were/are currently being used in Australia.

**Exclusion criteria (Round one)**

Studies were excluded if one or more of the following was determined:

- No data on the adverse effects;
- Study included paediatrics and we were unable to separately extract paediatric data;
- Duplicated studies;
- Study included only a single medication;
- Study population only included adults that were younger than 65 years;
- Primary objective was not the adverse effects of medications;
- Studies not in English;
- Studies focused on experimental medicines;
- Studies in phase III of a clinical trial.

**Exclusion criteria (Round two)**

- Model designs of the study were insufficiently described;
- Validation of tests were ambiguous;
- Designs and measures were not detailed;
- It was indeterminate as to whether the measure has been accepted in practice.

## 2. Methods

We designed and conducted our scoping review by following guidelines published by the Preferred Reporting Items for Systematic Reviews and Meta-analysis (PRIMSA) [7]. 

**Search strategy**

**Figure A1:**
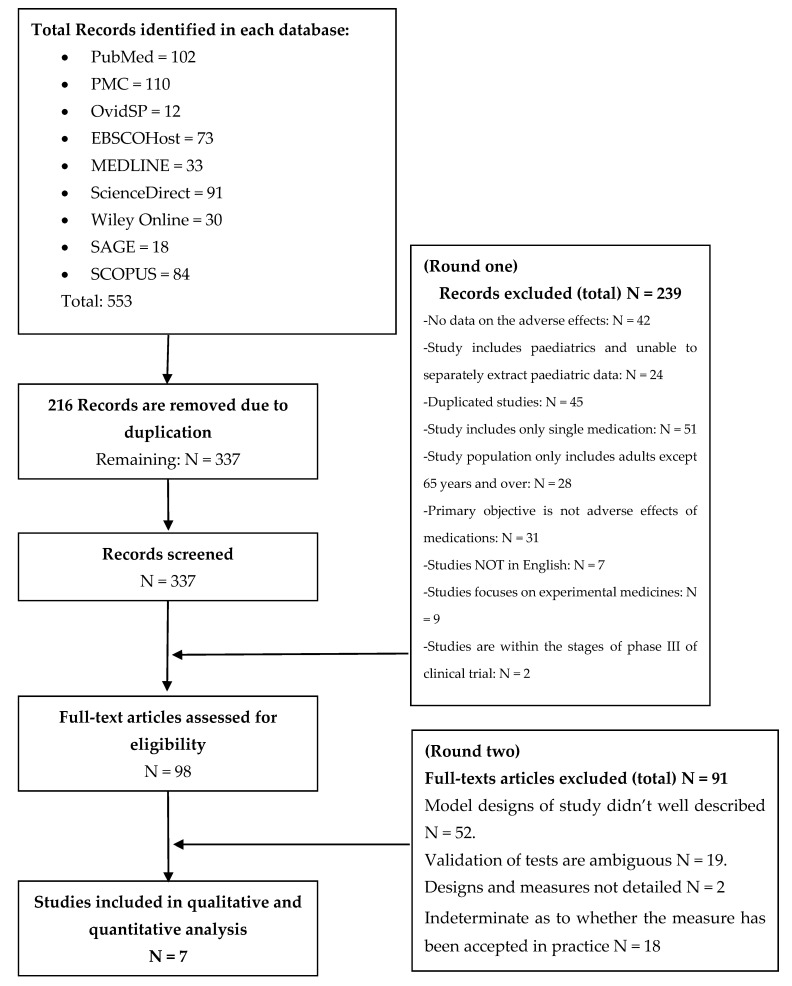
Implementation of search strategies and selection criteria.

The titles, abstracts, methods, results, discussion and finding for all published papers were screened by two reviewers against the agreed upon inclusion criteria. Disagreements between reviewers were resolved by further discussion of the reason for exclusion and a consensus was achieved. The search strategy and subsequent selection criteria of the identified published papers are displayed in Figure A1. A full search strategy for all databases is detailed in Appendix A. 

**Types of participants**

This review considered studies for the identification of the adverse effects that medications have had on older people in the primary care settings of Australia (regardless of whether the study was designed in Australia or overseas). Only studies that had their abstract in English were selected. There was no limitation in considering the date of acceptance for publication. 

**Concept**

This review explored and identified the characteristics of each study and critically measured their effectiveness on a patient’s health and wellbeing. Data from each study include: the number of participants in each study, drugs identified as contributing to major ADRs, rates of primary outcomes, drugs most frequently associated with outcomes, the most frequent body system affected by ADRs, acceptable low rates of loss to follow-up, binding outcome and potential bias.

**Context**

In this scoping review, no limit was set for the study setting or time frame. All studies, including the selected studies, were conducted in clinical settings (hospitals and nursing facilities). Table A1 is the summary of the selected studies.

**Information sources**

The database searches, up to February 2020, were obtained through PubMed, PMC, OvidSP, EBSCOHost, MEDLINE, ScienceDirect, Wiley Online, SAGE, and SCOPUS. Moreover, the searching strategy by Medical Subject Headings (MeSH) terms in popular and commonly used keywords and phrases was also obtained through PubMed. ScienceDirect and OvidSP searched for literature and dissertations, and abstracts were reached through SCOPUS. 

**Study selection**

The studies were identified through electronic databases and manual searches. A full set of the selected studies were exported from the databases into the reference manager software, EndNote X8 (Clarivate Analytics, PA, USA). Duplications were removed. Before formal screening and finalising the selection processes, a calibration exercise for the identified studies was performed by two reviewers (HQ and YG) independently. The purpose of this review was to refine the screening questions and to ensure consistency across reviewers for screening and to select eligible studies according to the inclusion criteria. Every article passed through a two-step process by two reviewers working independently: Step 1: the two reviewers (HQ and YG) screened all the titles and abstracts and they selected those that were relevant. Each reviewer independently assessed the article against the inclusion and exclusion criteria. The reason for exclusion was stated in EndNote. Step 2: after the abstract was selected, the full version of the selected article was retrieved and imported into EndNote. The two reviewers (HQ and YG) undertook a full review. Some studies were excluded after the selection. The reason for the exclusion of the full text review was noted in EndNote by each reviewer. The refined and retrieved articles for the review were compared by the two reviewers until the final agreed set was achieved. The disagreements between the two reviewers (HQ and YG) were resolved by mutual consensus discussion by a third co-author (MS or BD). None of the review authors were blinded to the journal titles, study authors, or institute where the article came from. The study selection process was determined and presented in the Preferred Reporting Items for Systematic Reviews and Meta-Analysis (PRISMA) flow diagram format. 

**Extraction and data presentation**

The data extracted from the included studies was based on the guides of the scoping review questions. The extracted data had been tabulated according to the author’s name, location, number of patients, number of drugs, rate of primary outcomes, drugs most frequently associated with outcomes, validation, most frequent body system affected by the adverse effects of medications, whether the selection was biased or not, acceptable low rate of loss to follow-up, and blinding outcome. Furthermore, the extracted data was audited and critically appraised by comparing data regarding their use in clinical practice, which health profession they were used by, if the study had been evaluated or not, which conditions were not used and why, and to determine if there were any limitations in practice. A summary table illustrating the audited and critically appraised data can be found in Table A2.

## 3. Results

The database searches yielded a total of 337 citations after duplicates were removed. The titles and abstracts for these 337 articles were screened by the first author, and 239 article titles and abstracts were excluded in round one due to having the following issues: the study had no data on adverse effects (42 articles), the study included paediatrics (24 articles), the study was duplicated (45 articles), the study used single medications only (51 articles), the study included participants younger than 65 years old (28 articles), the primary objective was not adverse effects of medication (31 articles), the study was not in English (seven articles), the study focused on experimental medicines (nine articles), and the study was in phase III of a clinical trial (two articles). The remaining 98 articles were considered for further detailed assessment of the full paper in round two, and 91 were excluded due to having the following issues: the model designs were not well described (52 articles), the methodologies were ambiguous (19 articles), the design and measures were not fully detailed (two articles), and the measure was not accepted in practice (18 articles). The search yielded a total of seven citations for inclusion in this review. 

**Outcome measured**

The seven studies that reported on the rate of adverse effects from prescribed medications in older people are summarized in Table A3. They identified which medications were involved in causing major adverse effects and worsened patient’s health conditions. However, none of these measures were able to predict the risk or rate of adverse drug effects to prevent health deterioration in older people. 

The cross-sectional study conducted by Harrison et al. [8] recruited 541 individuals from 17 residential aged care facilities around Australia. Of these, 82.8% were cognitively impaired and 64.3% were suffering from dementia. The objective of this study was to examine whether the DBI and Potentially Inappropriate Medications (PIM) were associated with quality of life in older people. This study was conducted with two instruments: the EuroQol Five-Dimensional Questionnaire (a measure of quality of life) and the Dementia Quality of Life Questionnaire. The results indicated that drugs with anticholinergic and sedative ADRs were associated with a lower quality of life [8]. 

Turner at al. [9] conducted a cross-sectional study to review the fall risk resulting from psychotropics and medications that cause orthostatic hypotension. This study involved 383 Australian older people whose medications were analysed with the Fall-Risk-Increasing Drugs (FRIDs) tool [9]. In comparison to older patients who were not frail, the outcome of this study identified that the risk of falls was underestimated or not recognised with respect to the contribution to risk for those drugs [9].

Inappropriate medication use is a common contributor to health deterioration in the elderly. Basger at al. [10] cross-referenced the treatment of common medical conditions in elderly people with the 50 highest-volume Australian Pharmaceutical Benefits Scheme (PBS) medications prescribed to Australians in 2006 [10]. The study found 96 cases that were not managed as effectively as they could be; 48 causes were dispensing overuse (e.g., too frequent use of medicines based on the prescribed dose). Eighteen patients who had a history of falls were not taking psychotropic medications (e.g., falls reported due to other medications rather than psychotropics, such as blood pressure medications). Nineteen patients with diabetes and cardiovascular events were not taking the recommended antiplatelet medicines or anticoagulants. Four patients taking non-steroidal anti-inflammatory drugs (NSAIDs) did not have pain. Three patients were taking additional selective serotonin re-uptake inhibitor (SSRI) together with other serotonergic effects and there were four cases of severe drug–drug interactions [10]. 

Ashoorian et al. [11] designed the My Medicines and Me Questionnaire (M3Q) as a self-reporting questionnaire for mental health patients who expressed concerns regarding side effects with their psychotropic medications [11]. A total of 205 older people from six mental health facilities were included. The results indicated that 77% reported sedation (a major risk of falls) and 23% reported gaining weight (a major risk of cardiovascular illness) [11].

The Modified Drug Adherence Work-Up Tool (M-DRAW) was developed by Lee et al. [12]. This tool has been designed to identify the barriers to medication adherence due to the side effects of medications. This tool asks the following “Do your medications give you side effects that make you NOT want to take it?” If so, further assessment of why the medications have side effects and the changed doses or changing medications will start from that point. This tool uses Likert scales for the responses (four-point scales of frequency) from one representing ‘never’ to four representing ‘often’. A pre- and post-interview design was established with a total of 172 participants. Based on their response, they were categorised into three adherence subdivisions: intentional non-adherence (INA), partial non-adherence (PNA), and adherers. Participants within INA and PNA groups were assigned to the intervention groups, while the adherer participants were assigned to the control group [12]. M-DRAW could provide recommendations to clinicians by giving them a systematic approach to overcome each identified barrier to adherence, especially non-adherence due to ADR [12]. 

McLeod et al. [13] developed a list of inappropriate prescribing practices for older people. The criteria were based on the following: prescriptions may introduce the patient to clinically significant risks of adverse effects, equally effective or more effective alternatives with less risk are available, and any clinical intervention that is reasonable enough to change the existing prescription to decrease morbidity [13]. The final list contained 71 inappropriate prescriptions for older people. Each item includes a clinical situation and each situation contains recommendations for alternative therapy and/or further investigations [13]. 

Finally, Nishtala and colleagues [14] conducted a drug burden index (DBI) study in 62 aged care facilities in New South Wales (NSW). DBI measures the effect of cumulative exposure to both anticholinergic and sedative medications on cognitive and physical functions in older adults [14]. DBI scores in older people were calculated, and the impact of the medication review on the DBI score after the uptake of pharmacist recommendations by GPs were evaluated. A total of 150,475 cases were collected (6751 cases including ADRs from psychotropic medications). The study determined and reported the neuropsychiatric adverse effects in older people [14]. 

## 4. Discussion

The current scoping review included a total of seven studies that met the inclusion criteria, so they investigated and described the adverse effects of the prescribed medication on older people by using tools or protocols designed for this purpose. For the Harrison study outcome, further studies would be suggested to examine whether deprescribing of medications included in the drug burden index (DBI) or Beers criteria may improve quality of life outcomes for these individuals, as well as to improve other consequences associated with reduced exposure to these medications, such as reduced hospitalisation and mortality [8]. 

Further studies are needed to establish the efficacy of the FRID tools and to rationalize or simplify medication regimens for elderly patients who are prescribed medications associated with orthostatic hypotension and psychotropics [9]. Further research will be required to determine whether de-prescribing fall risk-inducing medications will effectively reduce the risk of falls in older people [9]. 

Basger’s criteria was designed due to the deficiencies of the older Beers criteria in order to better suit the Australian health care system [10]. It is similar to Beers criteria, but it is a list of indicators based on, and derived from, Australian data sources rather than US sources [10]. The medications expressed in the collected sources have greater potential relevance in the Australian healthcare setting [10]. Additionally, it is developed from an analysis of the most commonly dispensed PBS medications and the most common conditions for which the elderly receive medical care [10].

M3Q has open-ended questions that elicit vital information about the patient’s adherence and evaluates the quality of life [11]. It allows the patients to communicate their feelings in writing by asking the patients to prioritise the most bothersome side effects. This instrument can also be used in metal health patients, but, in that case, the precision of the answers needs to be approved by nurses or doctors. As a result, it enables an action that would improve the therapeutic relationship with clinicians and improve adherence to prescribed medications [11]. Furthermore, this tool enhances clinician and patient communication and the capacity to work in partnership towards a common purpose. M3Q could be a subject of future investigations about variables that affect the patient’s perceptions and overall satisfaction with Australian heath care in a broader patient group [11]. 

The M-DRAW tool is acceptable and reliable to identify barriers to medication adherence and the causes behind non-adherence [12]. However, the validity of this tool is still uncertain, and further study needs to be done with a larger sample size and follow-up with patients [12]. 

The McLeod tool includes substantial information about the severity of the adverse effect of medications and rankings of the clinical importance of the medication risks [13]. The suggestions of alternative therapeutic options were based on the concept of more effective and less risky therapy [13]. This tool will help establish specific evidence-based guidelines for geriatric pharmacotherapy. Therefore, it would be advisable to revise the McLeod list of medications regularly, such as by further validation or validation in the Australian setting [13]. 

The findings by Nishtala’s study reinforce the importance of careful clinical assessment and management of older people who are at risk of increased anticholinergic burdens due to the use multiple neuropsychotropic drugs [14,15]. 

Generally, the idea of designing ADR assessment tools is essential at all stages of the medication management pathway. The designed tool needs to be derived from validated Australian data and be applicable to the Australian health care system. The designed tool needs to adopt the concept of multidisciplinary corporation, a structured approach to identify potential risks related to the risk of adverse effects of medicines and help to develop a framework for improvement strategies, and it can be a reliable resource to assist in reducing medication errors, overuse, and potential risky adverse effects. Thus, it may help clinicians to make the most appropriate clinical decisions for their patients.

## 5. Conclusions

To the best of our knowledge, numerous studies were done in Australia and overseas to assess the side effects of medication in older people. However, they are often specific to certain medical conditions and do not provide a comprehensive assessment of the medication’s adverse effects. This is of concern, given the increasing prevalence of age-related chronic diseases and associated disability, as well as the increasing number of Australians living in aged care facilities, leading to an increase in age-related disabilities and chronicity. Adverse medication-related incidents, unplanned medication related admission to hospital and inappropriate prescribing patterns are commonly observed in Australian elderly people. Moreover, these studies do not provide guidelines for alternative therapeutic options, nor do they provide recommendations that avoid interactions and ADRs. Therefore, it would be beneficial if Australian clinician researchers designed a predictive tool that integrates the information reported in this review to minimize the risks of ADRs. 

## Appendix A. Search Strategies

Our initial search used the syntax for MeSH terms (Medical Search Headings) in PubMed database as follows:

1. Medication adverse effects;
2. Elderly [all] OR Senior OR Older people [all] OR People aged over 65 OR [all] Geriatric OR Old;
3. Aging OR [all] Ageing OR Veteran [all] OR Older age;
4. Older people OR [all] older people OR [all] oldest people Older population;
5. Aged Care Facilities OR [all] Aged Care OR aged care OR [all] Nursing Home OR nursing facilities for aged care OR [all] nursing home OR Senior care [all] OR Care for older people OR [all] Care for advanced age;
6. Medication monitoring OR [all] Drugs monitor OR [all] Medicine monitor OR [all] medicine monitoring OR [all] therapy monitor OR curative monitoring OR [all] treatment assessment OR [all] therapy assessment OR remedy monitor [all] OR medication observation OR medication tracking OR [all] medicine records;
7. Drugs review OR Medication review OR [all] therapy regimen review OR [all] Medication check OR drugs check OR [all] medication rehearsal OR [all] medication revision OR [all] drugs revision OR [all] medication reassess OR [all] drugs reassessment OR [all] medication regimen appraisal OR [all] medication evaluation OR [all] drugs evaluation;
8. Adverse effects assessment OR [all] medication harm assessment OR [all] adverse effects revision OR [all] adverse effects evaluation OR [all] adverse effects evaluation OR [all] adverse effects estimation OR [all] adverse effects judgment OR [all] unwanted medication effects OR undesirable adverse effects OR [all] harmful medication effects OR [all] unfavourable drugs effects OR [all] pernicious drugs effects;
9. In Australia OR [all] In Australian heath care system OR [all] in Australian health setting;
10. #1 OR #3 and #5 and #6 and #7 and #8 OR 9# [all];
11. #2 OR #3 and #5 and #6 and #7 and #8 OR 10# [all];
12. #3 OR #4 and #5 and #6 and #7 and #8 OR 11# OR 12# [all].

The search strategy was developed and completed in PubMed, and the same strategy was then applied to the other databases (OvidSP, EBSCOHost, MEDLINE, ScienceDirect, Wiley Online, SAGE, and SCOPUS).

**Table A1:** Research Setting for the Selected Studies.

Included Study	Setting Research
Harrison et al., 2018—EuroQol questionnaire and DQL questionnaire study [8]	Australia aged care facilities and nursing home
Turner et al., 2016—FRIDs study [9]	Australian public hospitals and multi-disciplinary clinics
Basger et al., 2008—IMU-PI tool [10]	Australian health database
Ashoorian et al., 2015—M3Q Tool [11]	Australian mental health clinics and public hospitals
Lee et al., 2017—M-DRAW tool [12]	American-based study and used by Australian HMR †/RMMR ‡ Pharmacists
McLeod et al., 1997—McLeod Tool [13]	Canadian-based study and used by Australian HMR †/RMMR ‡ Pharmacists
Nishtala et al., 2009—DBI study [14]	Australian aged care facilities and nursing home

†. HMR: Home Medication Review; ‡. RMMR: Residential Medication Management Review

**Table A2:** Auditing and Critical Appraisal of Included Studies.

Tool	Current Use in Practice	Used by	Evaluation	When not Used and Why?	Limitations in Practice
DBI study (Nishtala et al. 2009)	62 aged care facilities in NSW. Determine DBI scores in older people in aged care homes; and evaluate the impact of RMMR on DBI score after uptake of pharmacist recommendations by GPs	Consultant Pharmacists in community and hospital settings, HMR^†^ and RMMR^‡^ accredited pharmacists	N = 500 residents, SD of age = 84.0 years, 25% male. SD for medications per resident = 7.4, SD for anticholinergic and sedative = 0.9 & 0.2 respectively. Reduction in prescribed anticholinergic and sedative medications can be achieved in older people through using DBI.	DBI is a formula designed to measure the adverse effects of anticholinergic and sedative medication on the quality of life. A higher DBI score represents a lower quality of life. DBI is not a tool for frequent use. It provides a reference for developing a RMMR report and subsequent pharmacist recommendations to GPs and nursing staff	This tool did not take into account differential pharmacokinetic properties of medications. No indication for drug–drug interactions provided and no pharmacodynamic profiles among aged care home residents are developed. No questionnaire; DBI calculations estimated as a liner dose-response relationship between drug classes. Predictive capacity of DBI not established. In this study the residents were not randomised into the intervention and control groups. This tool was applied retrospectively limiting any establishment of causality. No information about their health status or their disease severity was included.
INSPIRED study (Harrison et al. 2018)	Cross-sectional study: analysis of 541 individuals recruited from 17 aged care facilities in Australia (from NSW, QLD, SA, WA)	Nurses and carers in aged care facilities. This study was specific to older people living with cognitive impairment and dementia.	With respect to anticholinergic and sedative medications adverse effects, the PIM (Beers)^§^ criteria and DBI were highly prevalent in residential aged care at 73% and 83.1% respectively. Study confirmed higher exposure to these medications in inappropriate prescriptions were associated with a lower quality of life.	This study does not present a new tool. It is a comparison between DBI and PIM (Beer’s criteria) to determine whether these tools are associated with quality of life in older adults living in aged care facilities. It was only used in those with cognitive impairment and not for other medical conditions.	This study was unable to assess causality or the direction of any observed associated issues. In addition, these is no certainty of compatibility between the proxy measures that were used and what the individual would self-report if they able to do so.
FRIDs study Turner et al. 2016	Tertiary referral hospital in geriatric oncology outpatient multidisciplinary clinic.	Administrated by nurses, geriatricians, medical oncologists, geriatric oncology nurse, social workers, dietician, pharmacists, occupational therapists, and palliative care nurses.	Cohort study of older people with cancer. All data in this study verified by nurses with full access to patients’ medical records. Enabled inclusion of any omitted data to be collected. There was 79% concordance for self-reported prescribed medications compared with those obtained in an interview with clinical pharmacists in hospital wards.	Study limited to older people newly diagnosed with cancer, and previous history of falls / or orthostatic hypotension, and administrating psychotropic medications. Not applicable to older people administered psychotropic medications.	Single site data collection and not generalisable to other settings. Some patients did not know what fall was, others did not remember having fallen or they underreported the number of falls (if they fell several times). Not possible to determine if FRIDs study used at the time of fall or initiated after fall. In addition, the number of older people who received more than 3 prescribed medications of antipsychotic was small. These factors impacted the results of the adjusted multi-variate regression analysis giving wider confidence intervals.
IMU-PI tool Basger et al. 2008	Study tool design informed by expert’s review, international literatures, and clinical practice guidelines for medication use in elderly. The tool used with Australian heathcare system data and cross-referenced with treatment of common medical conditions for those with the highest volume of Australian Pharmaceutical Benefits Scheme usage in 2006 and 2007.	Experts from University of Sydney, NSW	Tool design is similar to Beers and McLeod tool. This tool had set out to develop an indicator list relevant to Australia the design did not involve an expert consensus process. Instead the tool was based on Australian healthcare data. Indicators had been selected from analysis of the most commonly dispensed PBS medications and based on the most common conditions for older people receiving medical care.	This study is NOT a specific tool or questionnaire used in age care facilities. This study performed only by collection of PBS data within only a two-year window. As a result, this tool has no ability to determine or detect the adverse effects of medications nor be used in any aged care facility.	The tool was not validated yet. This tool was not designed to act as a preventative health tool to avoid adverse events. It indicates that either appropriate or inappropriate medication has been prescribed.
M3Q Tool Ashoorian et al. 2015	Six public mental health clinics and one hospital in WA; 205 participants divided into intervention and control groups.	Nurses in mental health clinics	M3Q was designed specifically to assess the effects of antidepressants, antipsychotics, anxiolytic and mood stabilizers. This tool was developed to fill the gaps of lack communication between clinicians and patients. It contains closed and open response questions. It has been through rigorous validation processes; expert focus groups developed the design and psychometric testing. Focuses on patient’s list of self-reported medications and dose and they rank three bothersome side effects. Checklist of 32 possible side effects under 11 domains.	M3Q tool not applicable for older people suffering from other co-morbidities. The assessment of the psychotropic medication side effects does not reflect the reality of comorbidities and increases risk of inaccuracy.	This tool was not designed to objectively record the accurate number of psychotropic medications and their side effects. No statistically significant change was demonstrated within each group. M3Q tool was used a non-randomized convenience sample of patients. Many patients suffered other co-morbidities and were taking a number of medications not related to psychotropic medications or mental illness which may confound the assessment of side effects by clinician. A wider cross-section of patients attending GPs, pharmacies and wider representations would be worthwhile.
M-DRAW Tool Lee at al. 2017	Academic medical centre pharmacy in California-USA.	Pharmacists, nurses, social workers, and patient’s carers.	M-DRAW uses a motivational interview-based intervention strategy for each identified barrier. M-DRAW provides recommendations to clinicians on how to systematically approach follow-up for each identified barrier, and also identify the root cause of non-adherence.	This tool has been designed only for identifying barriers of medication adherence. It consists of a 13-item checklist questionnaire, and the results of this tool is scaled from 1 = never to 4 = often.	The limitation of this study was small sample size which limits generalisation. Test and re-test reliability were NOT performed, short duration study, follow-up items were not well defined. No specific illness dealt with; any chronic conditions. This tool assesses only non-intentional and intentional non-adherence of medications. This tool is applicable for a pharmacist conducting RMMR for medication-adherence assessment only.
McLeod Tool McLeod et al. 1997	Academic medical centres across Canada.	Pharmacists, doctors’ specialists, nurses, GPs, geriatricians.	New approach to identify inappropriate practice in prescribing medication for older people. This study has a list of 71 inappropriate practices in prescription for older people, and each practice rated from 1—not significant to 4 high significance. 3 major categories: drug contraindicated, drug-disease interactions and drug–drug interactions. The recommendation for each item could be generalizable.	This tool developed by Beers and collaborators resulting in considerable similarity between this tool and Beers criteria. This tool will be helpful for medication reviewing and preparing recommendations to GPs for consideration.	This study has no specific questionnaire and requires no interview with patients. It was designed only for detecting frequent inappropriate prescriptions for older people. The recommendation for each item was general with no further details or explanation.

†. HMR: Home Medication Review; ‡. RMMR: Residential Medication Management Review; §. PIM Beers criteria: Potentially inappropriate medicines

**Table A3:** Characteristics of Included Research.

Author	Location	No. of Patients	No. of Drugs	Drugs Most Frequently Associated with Outcomes	Validation	Most Frequent Body System Affected by ADRs	Selection was not Biased	Acceptability Low Rates of Loss to Follow-up	Blinding Outcome
Nishtala et al, 2009	Australia—database reported ADRs of psychiatric medications collected from TGA, PBS, health care professional (including hospitals and aged care facilities), consumers.	150475 (6751 cases and 123334 non-cases) Case is a report that include 1 or more neuropsychiatric ADRs. The non-case is a report that does not include any neuropsychiatric medication	Benzodiazepines, Anticholinergics, TCAs, and other 24 medications (CVDs, neurological, and pain management).	The following medications are results of 95% Cl for older + drug/older-drug. These medications producing more ADRs effects with older people than younger people: Cimetidine 2.24(1.7–3.0); Anticholinergic drugs 3.12(2.53-3.85); Antipsychotics 2.73(2.21–3.37); TCAs 2.31(1.93–2.77).	ADRs reports were validated by inclusion and exclusion criteria. Reports were excluded from the analysis if data for age or DOB were absent. Also, a combination of drugs including drug of interest had excluded as well from analysis. The 25 drugs of interest identified and assessed by Tune and coworkers.	CNS with major reports of agitation, anxiety, cognitive impairment, confusion, delirium, hallucinations, psychosis	The selection of report based on CNS signs such as history of hallucination, anxiety, agitation, depression, delirium and cognitive impairment. The association observed between drug exposure to the observed outcome may have been biased or distorted. Also, confounding by concomitant drug use gives concern of bias. In addition, the ADRs database are consist of reported adverse events information, thus subject to differential reporting are clearly biases. However, the authors minimised bias in this study by applying the drug of interest were not typically viewed as possessing anticholinergic characters. Furthermore, all reporting and coding had included in the analysis (not just those drugs that were coded as suspect drug for the reactions).	Not mentioned	Blinding was not reported in any stage.
Harrison et al, 2018	Australia—cross-sectional analysis of 541 older people recruited from 17 residential aged care facilities around Australia.	541	The criteria regarding drugs of interest based on Beers criteria and PIMs for all older people exposed for more than 8 weeks: PPIs 41.5%, Benzodiazepine 30.5%, Antipsychotics 24.8%, Antidepressants (mirtazapine 17.1%, sertraline 9.5%, escitalopram 8.6%, citalopram 7.1%), and Opioids (buprenorphine 14.3%, fentanyl 9.7%, oxycodone 8.2%). Benzodiazepine 9.9%. Antipsychotics (risperidone 12.7%).	Benzodiazepine, antipsychotics, antidepressants, and opioids	PIMs identified in this study by using validated measures of Beers criteria for older people. The facilities candidates have characteristic-levels were determined from information collected in a standardised questionnaire that was validated in older residential care population. This questionnaire includes 33 questions (asked about facility-level, location, No. of direct care hours per resident, size of facility, age, sex, marital status). The measures of EQ-5D-5L which completed by proxy has been validated in residential living in aged care facilities with dementia.	CNS and musculoskeletal system	Not mentioned	Not mentioned	Not mentioned
Turner at al, 2016	Australia, cross-sectional study at referral hospitals in Adelaide, geriatric oncology outpatients clinics.	383	Psychotropics, opioids, anxiolytics, hypnotics, sedatives, antidepressant, vasodilators in cardiac diseases, antihypertensives, diuretics, B-blockers, CCB, Renin-angiotensin system inhibitors, Alpha-antagonists, Dopaminergic agents.	Psychotropics, opioids, anxiolytics, hypnotics, sedatives, antidepressant, vasodilators in cardiac diseases, antihypertensives, diuretics, B-blockers, CCB, Renin-angiotensin system inhibitors, Alpha-antagonists, Dopaminergic agents.	This study is well-characterized cohort for older people with cancer. The validation stated from the initial appointment, and all data contained within the structured collection sheet which verified by nurses have access to participant’s medical records to allow any omitted data to be collected. Also, the validation of this study found that 77% concordance for self-reported prescription medication use when compared with participants obtained in an interview conducted by clinical pharmacist, which also comparable with medication has been taken routinely in hospital wards.	CNS and CVD	Not mentioned	Not mentioned	Not mentioned
Basger et al, 2008	Australia—cross-referenced treatment of the common medical conditions with the highest 50 PBS-medications prescribed to Australians in 2006.	50 highest used PBS-prescribed medications / documentations.	Top 50 prescribed medications in 2006 for Australian older people (>65 years old).	ACEI, ARB, Aspirin, B-adrenoceptor antagonists, Biphosphonates, Bupropion, Calcitriol, Calcium, Clopidogrel, Dipyridamole, inhaled corticosteroids, Intravaginal estrogen, Nicotine replacement medications, Paracetamol, Raloxifene, HMG-CoA (statins), Strontium, Teriparatide, Varenicline, Vitamin D, Warfarin.	The indicators of this study need to be tested and validated for relevance. However, the common anticipation is an identification of inappropriate medication use for commonly used medications in elderly Australians.	Heart failure, URI, depression, anxiety, arthritis, back pain, osteoporosis, falls, CVDs, renal impairment, GIT diseases (including GORD and ulcers), Type 2 diabetes mellitus, thyroid and parathyroid disorders, hepatic impairments, asthma and COPD, coagulation disorders.	Not mentioned	Not mentioned	Not mentioned
Ashoorian et al, 2015 (M3Q Tool)	Australia–adult people diagnosed with mental health conditions and they taking at least one or more psychotropic medications. Participants data collected from community and clinic public mental health services in west Australia.	205 patients: >50% male, Mean = 43 years, SD = 13. 73% reported taking multiple psychotropic medications.	All psychotropic PBS approvals	All psychotropic PBS approvals	M3Q tool was validated by provided participants an opportunity to express the impact of psychotropic medications side effects on their lives. Furthermore, the validation pf this tool passed through rigorous process: including eight focus groups with experts’ stakeholders to develop items followed by psychometric testing assessing the validity and reliability of the M3Q questionnaire.	Schizophrenia, bipolar disorder, depression, anxiety. These diseases usually associated with more or more comorbidities	Not mentioned	Follow-up after 3 months from the date of collection. Loss to follow-up had reported as 3 interviews abandoned to answer questions, 2 patients deceased and 14 decline to participate after 3 months from the first interview.	Not mentioned
(Lee at al. 2017) M-DRAW Tool	This study designed in USA-California and used in Australia. The study conducted in academic medical centres pharmacy in south California.	26	This study is non-drug focused. This study assesses factors contributing to medication non-adherence.	PBS-approved medication prescribed in chronic condition in adult and older people.	The validity been examined by applied pilot study of the psychometric properties of the M-DRAW tool to check the tool’s reliability. The validity of the tool was examined by priming question in 4-fold number of barriers to adherence within the self-selected intervention group and control group. However, confirmed validity not clearly stated because of small sample size and lost follow-up.	CVDs (hypertension and dyslipidaemia), type 2 diabetes, and chronic pain conditions.	Not mentioned	Loss follow-up reported in this study. For this reason, the validity of this study not been completely confirmed. The follow-up assessments were not collected as planned at the initial stage of the study protocol development because of short duration of this study time.	Not mentioned
McLeod et al. 1997 (McLeod Tool)	This study designed in Canada and using as a tool of older people I Australia. The participants from health professional of 32 specialities (7 clinical pharmacists, 9 geriatrics, 8 family GPs, and 8 community pharmacists).	32 health specialties recruited in academic medical centres across Canada.	CVDs drugs, psychotropic drugs, pain management drugs, and other miscellaneous drugs in older people.	B-blockers, AECIs, diuretics, CCB, benzodiazepine, TCA, barbiturate, antipsychotics, NSAIDs, phenylbutazone, warfarin, pentazocine, cimetidine, anticholinergics, antispasmodics, dipyridamole, diphenoxylate, cyclobenzaprine, methocarbamol.	Not mentioned	CVDs (including heart failure), asthma, COPD, mental issues (including dementia and insomnia), back pain, and osteoarthritis.	Not mentioned	Not mentioned	The collected list of inappropriate practice in prescribed medication underwent modifications before it is used in double-blinded controlled trial of a computer-based intervention for improving prescribing for older people.
